# Volatile Organic Compounds from Native Potato-associated *Pseudomonas* as Potential Anti-oomycete Agents

**DOI:** 10.3389/fmicb.2015.01295

**Published:** 2015-11-23

**Authors:** Mout De Vrieze, Piyush Pandey, Thomas D. Bucheli, Adithi R. Varadarajan, Christian H. Ahrens, Laure Weisskopf, Aurélien Bailly

**Affiliations:** ^1^Institute for Sustainability SciencesAgroscope, Zurich, Switzerland; ^2^Institute for Plant Production SciencesAgroscope, Wädenswil, Switzerland; ^3^Department of Microbiology, Assam UniversitySilchar, India; ^4^Swiss Institute of BioinformaticsWädenswil, Switzerland; ^5^CHANGINS, Viticulture and Oenology, University of Applied Sciences and Arts Western SwitzerlandNyon, Switzerland; ^6^Microbiology, Institute of Plant Biology, University of ZurichZurich, Switzerland

**Keywords:** *Phytophthora*, *Pseudomonas*, *Solanum tuberosum*, volatile organic compounds, biocontrol, microbiome

## Abstract

The plant kingdom represents a prominent biodiversity island for microbes that associate with the below- or aboveground organs of vegetal species. Both the root and the leaf represent interfaces where dynamic biological interactions influence plant life. Beside well-studied communication strategies based on soluble compounds and protein effectors, bacteria were recently shown to interact both with host plants and other microbial species through the emissions of volatile organic compounds (VOCs). Focusing on the potato late blight-causing agent *Phytophthora infestans*, this work addresses the potential role of the bacterial volatilome in suppressing plant diseases. In a previous study, we isolated and identified a large collection of strains with anti-*Phytophthora* potential from both the phyllosphere and the rhizosphere of potato. Here we report the characterization and quantification of their emissions of biogenic volatiles, comparing 16 *Pseudomonas* strains differing in (i) origin of isolation (phyllosphere vs. rhizosphere), (ii) *in vitro* inhibition of *P. infestans* growth and sporulation behavior, and (iii) protective effects against late blight on potato leaf disks. We systematically tested the pharmacological inhibitory activity of core and strain-specific single compounds against *P. infestans* mycelial growth and sporangial behavior in order to identify key effective candidate molecules present in the complex natural VOCs blends. We envisage the plant bacterial microbiome as a reservoir for functional VOCs and establish the basis for finding the primary enzymatic toolset that enables the production of active components of the volatile bouquet in plant-associated bacteria. Comprehension of these functional interspecies interactions will open perspectives for the sustainable control of plant diseases in forthcoming agriculture.

## Introduction

After more than a decade of exploratory work, it is now recognized that beside their well-documented, soluble anti-microbial arsenal, bacteria emit a wide range of volatile organic compounds (VOCs) that hold a strong inhibitory potential against microbial competitors (Garbeva et al., 2014; Audrain et al., 2015; Hunziker et al., 2015; Kanchiswamy et al., 2015; Schmidt et al., 2015). Therefore bacterial VOCs (bVOCs) are currently bringing an additional motivation to prospect the plant-associated microbiome with respect to its ability to confer to crop plants a natural protection against microbial pathogens. Indeed, *in natura*, these molecules are suspected to mediate or participate in intra- and interspecies communication processes such as bacterial quorum sensing, growth, differentiation or antibiotic and stress resistance (Vespermann et al., 2007; Bailly and Weisskopf, 2012; Bos et al., 2013; Groenhagen et al., 2013; Audrain et al., 2015; Kanchiswamy et al., 2015). Next, under laboratory conditions, bVOCs were also demonstrated to hinder growth and differentiation in numerous phytopathogenic fungal species (Kai et al., 2007; Athukorala et al., 2010; Ting et al., 2011; Velazquez-Becerra et al., 2011; Effmert et al., 2012; Yuan et al., 2012; Groenhagen et al., 2013; Tenorio-Salgado et al., 2013; Hunziker et al., 2015), suggesting that the complex blends of bacterial emissions represent a source of novel, naturally produced, anti-fungal substances. Finally, plants themselves were shown to directly react to microbial volatiles, resulting in direct or indirect plant health and growth promotion (Vespermann et al., 2007; Zhang et al., 2007; Kai et al., 2008; Gutierrez-Luna et al., 2010; Kai and Piechulla, 2010; Kwon et al., 2010; Blom et al., 2011a; Bailly and Weisskopf, 2012; Bitas et al., 2013; Farag et al., 2013; Weisskopf and Bailly, 2013; Bailly et al., 2014). Taken together, these insights interrogate the role of the bacterial volatilome in the dynamic interactions taking place in the plant natural environment. The plant-associated microbiome is virtually covering the entire plant surface and is especially abundant in the nutrient-rich rhizosphere, where the competition pressure between organisms is high (Berendsen et al., 2012; Mendes et al., 2013; De-la-Peña and Loyola-Vargas, 2014). On aerial organs, specialized bacteria survive in hostile niches in close-association with the leaf tissues (Vorholt, 2012; Bulgarelli et al., 2013; Junker and Tholl, 2013). On both below- and aboveground plant structures, bacterial populations have thus the possibility to convert metabolites found in the environment into volatile effectors, which are expected to be particularly effective when in close range of pathogens’ invasion points. The pathosystem *Phytophthora infestans*-potato represents a good model to investigate VOCs’ contributions to the microbial relationships occurring at the plant soil and air interfaces. This devastating pathogen, which causes the economically highly relevant potato late blight disease, can both infect aerial and soil organs. Furthermore, it enters the plant tissues where bacteria have the greatest potential to reside: around the root cap, on the leaf surface and in the stomatal chamber (Fry, 2008). In a previous screen for the anti-oomycete potential of potato-associated bacteria natively present in the rhizo- and phyllosphere, we isolated and characterized 16 *Pseudomonas* strains with various degrees of VOCs-mediated efficacy against *P. infestans* radial mycelial growth. Our work advocated for the existence of effective bVOCs against *P. infestans* on top of well-recognized potent inorganic compounds such as hydrogen cyanide or ammonia (Voisard et al., 1989; Rudrappa et al., 2008; Blom et al., 2011b; Hunziker et al., 2015). Although many bacterial volatile compounds have been reported as bioactive against pathogens (Vespermann et al., 2007; Athukorala et al., 2010; Ting et al., 2011; Velazquez-Becerra et al., 2011; Effmert et al., 2012; Yuan et al., 2012; Groenhagen et al., 2013; Tenorio-Salgado et al., 2013; Wang et al., 2013; Hunziker et al., 2015), a large majority of the available literature has reported the effects of a few prominent molecules recorded from a limited number of bacterial strains only, if not single isolates. We previously followed the same logic and reported the main *Pseudomonas* sp. volatile metabolite 1-undecene as an active ingredient of the anti-oomycete properties of eight *Pseudomonas* isolates’ volatilome (Hunziker et al., 2015). However, treating *P. infestans* with this single compound did not reach the full inhibition capacity of natural VOCs blends, suggesting that more volatile molecules are involved in the anti-oomycete activity of the *Pseudomonas*. Here we report the gas chromatography-mass spectrometry chemoprofiles of 16 selected *Pseudomonas* strains and the systematic testing of the activity of their individual pure chemical components against the growth and sporulation of *P. infestans*. Our goal was to determine the contribution of each chemical species present in the recorded bVOC spectra to the inhibition of *P. infestans* and consequently try to identify specific compounds or chemical families required for the anti-oomycete activity. Our results suggest that, in addition to biogenic soluble chemicals or protein effectors, the quest for bacterial bio-control agents should take into account the enzymatic traits leading to the production of VOCs as they represent a supplementary defense line against infection by plant pathogens.

## Materials and Methods

### Chemicals and Culture Media

Chemicals were purchased from Sigma–Aldrich (Switzerland) with the exception of 1-dodecene (Dr. Ehrenstorfer GmbH, Germany) and 2-acetylfuran (Alfa Aesar, Germany). Luria-Bertani (LB) medium was prepared by dissolving 20 gl^-1^ of Difco LB Broth, Lennox (BD) and adding 15 gl^-1^ agar (Agar Agar, ERNE surface AG). Rye agar (RA) was prepared by gently boiling 200 g rye grains in 1.5 l tap water for 1 h. The liquid was then filtered through a sieve (1.5 mm mesh) and filled up to the end volume of 1 l with tap water and supplemented with 5 gl^-1^ D-glucose. 20 gl^-1^ agar were added. Petri dishes were filled using a plate-pouring machine (Mediajet, Integra Biosciences) with 18 ml of medium in standard Petri dishes (94 mm × 16 mm, Greiner Bio-One).

### Microbial Strains and Culture Conditions

A *P. infestans* polypore isolate sampled in 2001 (provided by H. Krebs, Agroscope) was used for all experiments. This isolate had been maintained as mycelial culture on RA and regularly transferred to potato slices for host passage. Petri dishes were sealed with Parafilm M (BEMIS Flexible Packaging) and incubated or stored in the dark at 18°C.

Most bacteria were isolated and maintained as in (Hunziker et al., 2015). *Pseudomonas protegens* CHA0 and its corresponding *hcn*^A-^ mutant CHA77 were obtained from Prof. Dr. Dieter Haas (University of Lausanne). Bacterial strains were routinely grown on LB and kept at ^-^80°C in 25% glycerol for long-term storage.

### Multi Locus Sequence Alignments

In order to elucidate the phylogenetic relationships of the 16 candidate *Pseudomonas* sp. strains with respect to additional selected *Pseudomonas* reference strains, the sequences of four major housekeeping genes including 16s rRNA, gyrB, rpoD, and rpoB (Mulet et al., 2010; Gomila et al., 2015) were extracted from an Illumina MiSeq paired end (2x 300 bp) sequencing effort aimed at describing the gene inventory of these strains. The contigs that resulted from *de novo* genome assembly using the Spades algorithm (Bankevich et al., 2012) were then annotated with the software Prokka (Seemann, 2014), prior to retrieving the sequences of the four housekeeping genes and subsequently concatenating them in the order 16s rRNA, gyrB, rpoD, and rpoB. The MEGA (Molecular Evolutionary Genetic Analysis) software (Kumar et al., 2008) was used to construct a maximum-likelihood-based phylogenetic tree from the concatenated sequences of the four genes of 16 strains, as well as of nine additional reference *Pseudomonas* strains, including *P. protegens* CHA0. All generated sequences were deposited to GeneBank under the accession numbers KT890284 - KT890343^1^.

### Collection of Volatiles and GC/MS Analysis

The volatiles of sixteen selected strains were collected and analyzed by gas chromatography-mass spectrometry (GC/MS) using closed-loop-stripping analysis (CLSA) as described earlier (Hunziker et al., 2015) using a modified apparatus design (see Supplementary Figure S5). The strains were pre-grown at 18°C on LB-agar plates for 4 days before single colonies were resuspended and adjusted to a density of OD_570nm_ = 1 in sterile water. Bacterial samples were cultured by inoculating 50 μl of cell suspensions, spread as a layer using sterile glass beads into 4 cm glass Petri dishes to avoid plastic volatile contaminants and grown for 24 h at 25°C in the CLSA apparatus under constant air flow. Uninoculated LB-agar glass plates were used as controls. Trapped volatiles were extracted from the charcoal filter by rinsing the filter three times with 20 μl dichloromethane (≥99.8%, Merck, Germany). The headspace extracts were subsequently adjusted to 100 μl with dichloromethane and analyzed by GC/MS. Each experiment was repeated three times. Analyses by GC/MS were performed on a Varian CP3800 gas chromatograph (Varian, Walnut Creek, CA, USA) connected to a triple quadrupole mass spectrometer (Varian 1200, Varian). Separation by GC took place on a Rtx-5Sil MS capillary column (30 m, 0.25 mm i.d., 0.25 μm film thickness) from Restek (Bellafonte, PA, USA). As a retention gap, a 2 m Siltek guard column (0.53 mm i.d., Restek) was mounted in front of the separation column. Helium was used as a carrier gas at a constant flow of 1 ml.min^-1^. The samples were injected on-column into a programmable temperature vaporization injector (temperature program: 50°C for 0.1 min, to 300°C at 200°C.min^-1^, 30 min at 300°C, to 160°C at 20°C.min^-1^, 20 min at 160°C). The oven temperature was programmed as follows: 10 min at 40°C, to 320°C at 25°C.min^-1^, 1 min at 320°C. The transfer line temperature was set at 200°C. Analyte detection by MS was conducted in the electron impact mode with 70 eV ionization energy, at a source temperature of 250°C. Full scan monitoring (scan time 0.7 s) was performed in the m/z mass range from 35 to 350. Compounds were identified by comparison of mass spectra to database spectra (NIST 08 and pure commercial reference compounds), and comparison of the retention times and mass spectra previously obtained (Hunziker et al., 2015).

### Effect of Pure Compounds on *P. infestans* Mycelial Growth and Sporulation

The effect of selected pure compounds of the VOCs blends on mycelial growth, sporangia production and germination, zoopore release, motility and germination of *P. infestans* was assessed as follows: 5 mm agar plugs from the edge of actively growing mycelial colonies were placed downward-faced in the center of fresh RA plates. Definite quantities of the test compounds or dilutions in dimethylsulfoxide were applied on PTFE/silicone septa (8 mm, Supelco) and placed in the center of the Petri dish lid so the test compounds faced the mycelial plug. Plates were sealed with Parafilm M and incubated upside-down in the dark at 18°C. Mycelial growth was monitored 7 days after inoculation by taking photographs and total mycelial area was further assessed using ImageJ. At the end of a 9-day incubation period, the plates were opened and sporangia were collected using 5 ml room temperature sterile water by gently rubbing the mycelial mat with a sterile glass rod. A 500 μl aliquot of the resulting suspension was dispensed into a 24-well polycarbonate plate. The number of produced sporangia and rate of germination was assessed under the binocular right after collection and 24 h later, respectively. The same procedure was used to produce zoospores, except that 10 ml of ice-cold water was laid on top of the mycelial mat and kept at 4°C for 2 h. After this incubation period, the suspension was left at room temperature for 20 min prior to collection in order to allow zoospore release to occur. A 500 μl aliquot of the resulting suspension was dispensed into a 24-well polycarbonate plate. The quantity of produced zoospores and zoospore motility was assessed under the binocular right after collection. Zoospore encystment and subsequent germination was assessed 24 h later.

To evaluate the effect of pure compounds on direct sporangial germination, sporangia were collected in 10 ml room temperature sterile water from 9 days-old mycelial plates and adjusted to a concentration of 200’000 sporangia.ml^-1^ in Eppendorf tubes supplemented with the adequate chemical treatment. No vortexing was applied in order to avoid triggering germination. After 1 day of incubation at 20°C in the dark, series of 10 μl droplets of the suspension were mounted on glass slides and photographed under the microscope. The number of closed, open and germinating sporangia were counted using ImageJ. In both experimental setups, the inhibitory concentration yielding 50% inhibition (IC_50_) of mycelial growth or sporangial germination for each treatment was calculated through extrapolation from the curve-fitted plots.

### Zoospore Tracking

Zoospores from preparations obtained as described above were used to follow the trajectories of individual cells over time. Image sequences were taken for 10 s, using a Wild Heerbrugg MDG17 binocular (Wild Heerbrugg, Switzerland) coupled to a Leica DFC290 Camera using the LAS software v4.6.2 (Leica, Germany). Images were next filter-transformed using ImageJ to achieve high contrast. Single particle tracking over the segmented images was performed using the ImageJ TrackMate plugin^2^, (Jaqaman et al., 2008) using semi-automated default parameters. Chemical treatments were performed immediately prior to observations and image capture to avoid any bias from the decrease of zoospore motility occurring in control conditions.

### Fluorescent Label Microscopy and Analysis

*Phytopthora infestans* 208 m^2^ was obtained from Prof. Dr. F. Mauch (University of Fribourg) and maintained on RA plates as described above. Sporangial yield was increased by exposing the Petri dish to natural light for 8 h at days 7 and 8 after inoculation. Sporangia were collected as described above 9 days after inoculation and GFP fluorescence was visualized and digital micrographs acquired using a Zeiss Axiovert 200 M stereomicroscope equipped with adequate fluorescent source and filters. The GFP signal mean gray intensities of individual sporangia were extracted using a custom-made segmentation macroinstruction protocol to estimate the signal intensity distribution at the population level.

### Leaf Disk Assays

A 10 μL drop of a water suspension of 125’000 sporangia.mL^-1^ was applied in the middle of the abaxial side of a ø17 mm leaf disk cut from 1 month old potato plants *cv.* Victoria. Leaf disks were placed on a soaked filter paper in standard Petri dishes. Definite amounts of the test compounds were applied on PTFE/silicone septa (8 mm, Supelco) and placed in the center of the Petri dish, 3 cm away from each leaf disk. Petri dishes were closed with Parafilm M, placed in a lightproof plastic box and incubated at 18°C for a period of 8 days. *P. infestans* infection was then visualized and acquired under the binocular.

### Data Analysis

Data were analyzed using the GraphPad Quikcalcs tools^3^, GraphPad Prism 5 software and Microsoft Excel software.

## Results

### The Plant-associated *Pseudomonas* Chemoprofiles Present a Conserved Volatilome

**FIGURE 1:**
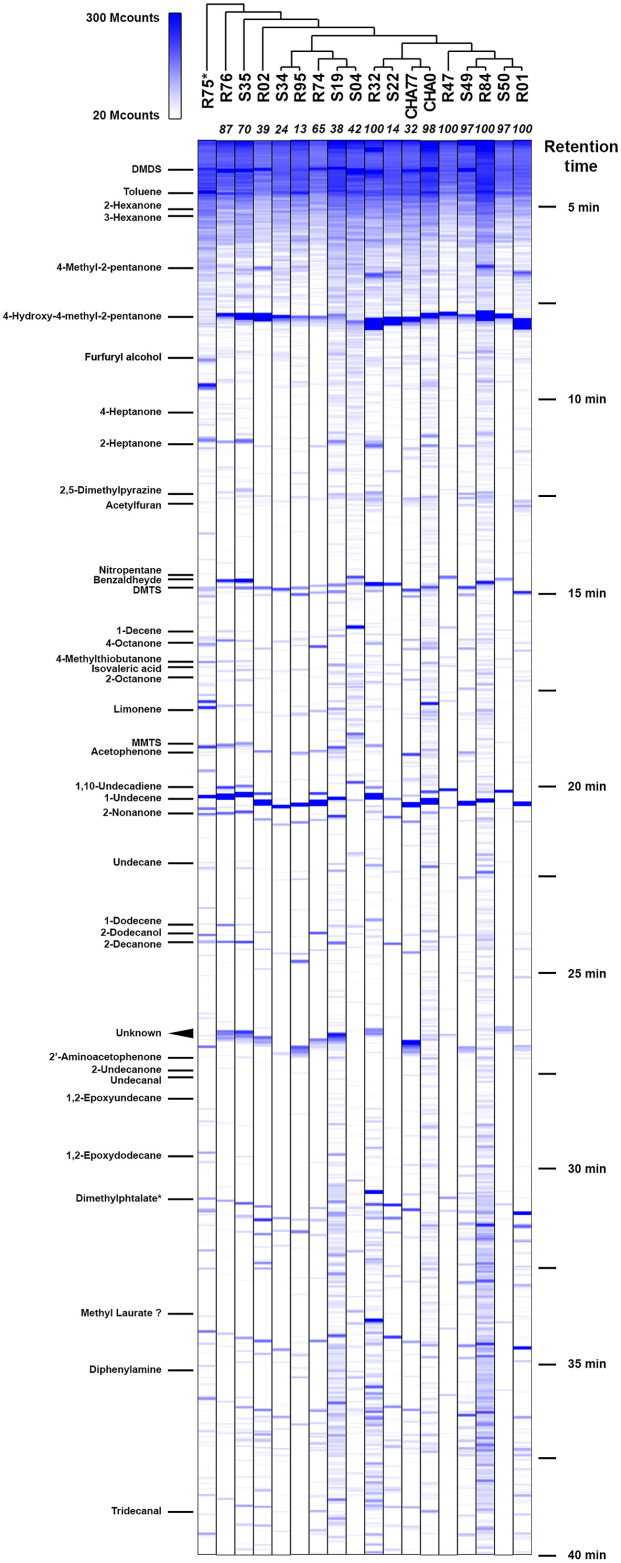
**Chemoprofiles of volatile organic compounds (VOCs) emitted by potato-associated *Pseudomonas*.** Representative gas chromatography spectra for each strain are ordered according to phylogenetic clustering from multilocus sequence alignments (Supplementary Figure S2). Selected identified compounds are indicated on the left; their approximate retention times can be deduced from the right legend. Numbers in italics at the top of each spectrum represent the percentage of *Phytophthora infestans* growth inhibition triggered by the strains’ VOCs blends. MS signal intensities are expressed in megacounts (Mcounts). ^∗^R75 was not identified as a *Pseudomonas* strain.

In order to correlate the production of specific bVOCs to the inhibitory potential of the 16 different *Pseudomonas* strains we previously screened, showing various VOCs-mediated inhibition against *P. infestans* (Hunziker et al., 2015), we trapped and identified the different chemical species they emitted. With the underlying hypotheses that the most active strains should produce either (1) a different set of compounds or (2) differential amounts of specific compounds, we cultured and further analyzed the bacterial emissions in standardized conditions. All tested strains were inoculated with the same density of cell suspension and let grow for the same amount of time under identical culture conditions. We chose to collect the bVOCs over a 24 h incubation period (i.e., until late stationary phase) with the aim to maximize the chance of recovering chemoprofiles that would best represent the *P. infestans* growth-inhibiting bVOC blends occurring in our previously published dual-assays (Hunziker et al., 2015). Furthermore, a steady closed-loop-stripping apparatus allowed us to trap and extract the emitted chemical species with high reproducibility. Consequently, although the GC/MS spectra obtained do not report quantitative amounts of individual chemical species, they capture very comparable relative profiles of the different test strains’ biogenic emissions (**Figure 1** and Supplementary Figure S1).

**FIGURE 2:**
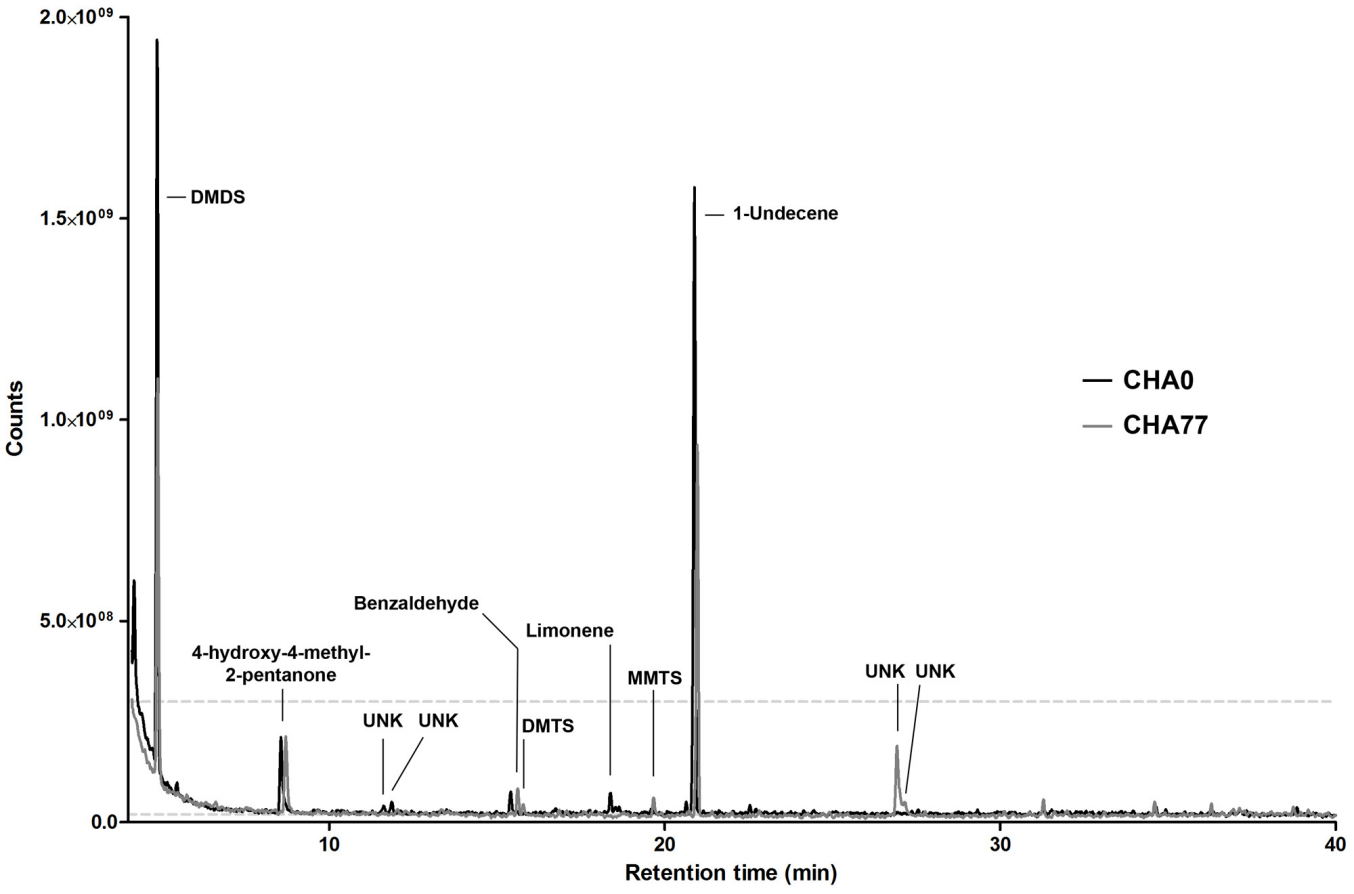
**Representative gas-chromatograms of *Pseudomonas protegens* CHA0 and its HCN-deficient mutant CHA77.** Major peaks and associated compounds are indicated. UNK, unknown chemical species.

Under these conditions, we were able to retrieve a large majority of the volatile compounds identified earlier and obtained similar chromatograms (Hunziker et al., 2015). However, slight changes in the gas chromatograph setup and parameters allowed us to get a better insight into the smallest and more hydrophilic components of the volatile emissions from plant-associated *Pseudomonas*. To our surprise, we discovered that in many of the tested strains the sulfur-containing compound dimethyldisulfide (DMDS) was produced in similar or higher amounts as 1-undecene (**Figure 1**), which we have described earlier as being the major chemical in the bacterial VOCs mixture (Hunziker et al., 2015). When GC peak area of definite amounts of pure DMDS and 1-undecene where compared to test spectra, a 24 h collection period yielded micrograms of both compounds, thus underlining the capacity of bacterial strains to accumulate high concentrations of VOCs in the headspace. In the vast majority of strains, 1-undecene, DMDS, 4-hydroxy-2-pentanone and benzaldehyde were the major peaks of the emission spectra. No particular chemical patterns could be unequivocally found between the different strains included in this study, neither by clustering the spectral data according to the strains’ phylogeny (**Figure 1**, and Supplementary Figure S2) or origin of isolation (rhizosphere vs. phyllosphere, Supplementary Figure S1). We also did not observe a straight correlation between the presence or the amounts of a given chemical species and the full VOCs blends effects of the strains on *P. infestans* radial growth (Hunziker et al., 2015, **Figure 1**). While the strongest *P. infestans* growth inhibition was as expected caused by HCN-producing strains (Hunziker et al., 2015), the individual volatile compounds that could contribute to the *P. infestans* inhibition triggered by non-HCN producing strains were not obvious to deduce. However, comparison of the emission spectra of the biocontrol *P. fluorescens* CHA0 (Voisard et al., 1989) with those of its corresponding HCN deficient, *hcn*A^-^ mutant CHA77 (Laville et al., 1998) revealed differences in the metabolite production (**Figure 2**). Indeed CHA77 produced lower amounts of DMTS and 1-undecene, and significantly higher amounts of DMTS and MMTS. In addition, we observed a set of two or more yet uncharacterized compounds (rt = 26.8 min) that seem to be common products from non-cyanogenic bacteria (**Figure 1**). Likewise, the CHA0 strain produced higher amounts of limonene and two supplementary compounds that await formal identification. From the same initial inoculum density, several strains such as R32, R84, CHA0, S04, or S19 had apparent higher levels of volatile production that could be explained by a higher metabolism or a better adaptation to the culture conditions (**Figure 1**). Nevertheless, the phylogenetic group comprising R74, R95, S04, S19, and S34 seemed to have repeatedly produced smaller amounts of 4-methyl-2-pentanone and 4-hydroxy-4-methyl-2-pentanone. Moreover, strains that produced the lowest, or not detectable amounts of DMDS, R01, R32, R47, S22, and S34, showed also the lowest detected amounts of DMTS and MMTS. Finally, the strain R75, that displayed a clearly dissimilar chemoprofile, was formally identified as belonging to the *Flavobacterium* genera, and not as previously reported to *Pseudomonas* (Hunziker et al., 2015), supporting the concept that bacteria could be identified at the species level via their volatile chemical signature (Thorn et al., 2011).

### Effects of Pure Volatile Compounds on *P. infestans* Growth and Sporangial Behavior

The high number of sampled *Pseudomonas* volatile profiles and their high similarity prompted us to investigate the contribution of a subset of individual chemical species to the previously observed growth inhibition of *P. infestans* by VOCs inhibition. Although not exhaustive, the list of tested, commercially available compounds we present here covers a large portion of the recorded *Pseudomonas* spectra (**Figure 1** and **Table 1**). While our pharmacological screen revealed that half of the tested compounds showed mild to low growth inhibition of the plant pathogen at high applied amounts, only few compounds possessed strong inhibitory activity (i.e., an IC_50_ below 1 mg, reaching at least 30% inhibition and a *R*^2^ > 0.6) against *P. infestans* mycelial growth. Nitropentane, isovaleric acid, undecanal, phenylpropanedione, propiophenone, dimethyl trisulfide (DMTS) and *S*-methyl methanethiosulfonate (MMTS) showed total inhibition of *P. infestans* growth at 1 mg. Although 1-undecene did not perform very well in hindering *P. infestans* growth within the applied amounts, its inhibitory activity was found to be close to those reported earlier (Hunziker et al., 2015). In our hands, the bacterial quorum-sensing-related molecules 2’-aminoacetophenone and acetophenone had low influence on growth of the pathogen, reaching merely about 30% mycelium area reduction. In contrast, cyclic ketones sharing a similar backbone such as propiophenone, and phenylpropanedione were amongst the most potent chemicals assayed in this study, with IC50 values ranging from 10 μg to 0.5 mg. Strikingly, nearly all sulfur-containing VOCs showed a great mycelial growth inhibition potential. From all assessed chemical species, MMTS was the most potent with an estimated IC_50_ in the low nanogram range. We did not observe specific apparent phenotypic defects in *P. infestans* upon exposure to the tested pure chemicals, with the exception of 2-undecanone (and to a lesser extent 2-decanone) treatments, which resulted in a dose-dependent densification of the mycelial mat and extended mycelium aerial growth, resulting in a “fluffy” appearance of the colony (Supplementary Figure S3).

**Table 1:** *Phytophthora infestans* mycelial growth inhibition after exposure to pure volatile organic compounds (VOCs).

	No constraints				Constrained fit Top = 100, Bottom= 0^a^	
	Bottom^a^	Top^a^	Rel. IC50 (mg)	*R*^2^	IC50 (mg)	*R*^2^
**Nitropentane**	**-5.583**	**98.73**	**0.02563**	**0.9719**	**0.02163**	**0.9682**
Undecane	114.1	100.9	0.01501	0.2055	^∗^	^∗^
1,2-Epoxydodecane	39.74	90.44	2.51	0.2489	2.911	-0.3972
4-Hydroxy-4-methyl-2-pentanone	113.4	99.79	0.000272	0.122	42.59	-0.3338
2-Hexanone	69.61	99.21	0.000292	0.4418	2.809	-1.027
3-Hexanone	^∗^	^∗^	^∗^	^∗^	^∗^	^∗^
2-Heptanone	∼-14680	98.32	∼418.9	0.4586	1.754	0.446
4-Heptanone	^∗^	^∗^	^∗^	^∗^	^∗^	^∗^
**2-Octanone**	**59.42**	**99.61**	**9E-05**	**0.7706**	**0.3907**	-**1.197**
**4-Octanone**	**41.59**	**95.91**	**0.02942**	**0.8831**	**0.1871**	**0.4772**
2-Decanone	90.9	99.99	2.55E-05	0.1651	4.735	-0.02398
2-Undecanone	71.13	99.58	0.000398	0.3975	3.126	-0.807
4-Methylthiobutanone	-969900	100.1	47231	n.d.	3.961	0.2865
*S*-Limonene	132.5	100.1	0.6097	0.4483	^∗^	^∗^
*R*-Limonene	^∗^	^∗^	^∗^	^∗^	^∗^	^∗^
1-Undecene	75.48	98.87	0.000207	0.6268	1.739	-0.6645
1-Dodecene	^∗^	^∗^	^∗^	^∗^	59.14	-0.1474
**Isovaleric acid^†^**	-**62.37**	**103.9**	**0.8344**	**0.8724**	**0.3167**	**0.8337**
Methyl laurate	^∗^	^∗^	^∗^	^∗^	3.565	-2.136
**Undecanal**	-**5.856**	**106.3**	**0.01611**	**0.9688**	**0.01638**	**0.9562**
Tridecanal	73.23	105.6	0.001336	0.6605	3.035	-0.2182
Acetylfuran	71.97	99.26	0.000151	0.5422	1.034	-0.09331
Diphenylamine	^∗^	^∗^	^∗^	^∗^	^∗^	^∗^
*S*-Methylbutanethioate	∼-5331	99.95	∼102.9	0.7277	0.9715	0.7188
Bis(methylthiomethyl)sulfide	90.34	94.02	0.04948	0.004254	8.637	-0.07327
Dimethyl disulfide	∼-13740	96.01	∼559.2	0.6117	2.452	0.5208
**Dimethyl trisulfide**	-**9.093**	**99.84**	**0.06971**	**0.8518**	**0.0574**	**0.8443**
***S*-Methyl methanethiosulfonate^†^**	**1.561**	**93.72**	**0.004302**	**0.9854**	**0.003588**	**0.9775**
Dimethylpyrazine	81.92	100.5	5.56E-05	0.3498	4.979	-0.4588
2-Dodecanol	81.48	93.01	0.2443	0.1974	4.7	-0.5748
Furfuryl alcohol	73.75	103.9	0.000571	0.5662	2.456	-0.318
Farnesyl acetone	83.45	99.95	3.27E-05	0.3287	2.512	-0.3345
2′-Aminoacetophenone	**69.61**	**100**	**1.06E-05**	**0.6025**	**2.043**	-**2.174**
Acetophenone	74.4	99.56	0.000218	0.4854	1.147	0.1975
**Phenylacetone**	**60.35**	**99.42**	**9.66E-05**	**0.7837**	**0.4146**	-**1.168**
**Propiophenone**	-**3.049**	**99.82**	**0.02381**	**0.9769**	**0.02195**	**0.9758**
**Phenylpropanedione^†^**	-**7.092**	**106.5**	**0.01691**	**0.9765**	**0.01677**	**0.9618**
**2-Phenylethanol**	**43.46**	**96.46**	**0.02484**	**0.8582**	**0.1679**	**0.36**
2-Acetylthiazole	^∗^	^∗^	^∗^	^∗^	^∗^	^∗^
Benzaldehyde	^∗^	^∗^	^∗^	^∗^	70.75	-0.1969

*^a^% of P. infestans mycelial growth; ^†^Compounds considered efficient in both mycelial growth and sporangial germination inhibition; ^∗^Curve fitting did not converge. Grayed cells indicate S-containing compounds. Relevant non-linear regression parameters [Log(inhibitor) vs. Response] are derived from pharmacological assays. All test compounds were assayed at 1 mg, 100 μg, 10 μg, 1 μg, 100 ng. In addition, DMTS was tested at 5 mg, 500 μg and 50 μg and dimethylpyrazine and MMTS were tested at 2 mg, n = 4–12. Curve fitting was performed with or without maximal constraints (top = 100% of P. infestans growth, bottom = 0% of P. infestans growth) to extrapolate IC_50_ and relative IC_50_ values for each compound, respectively. Bold indicates compounds displaying at least 30% inhibition, an IC_50_ below 1 mg and a *R*^2^ > 0.6.*

*Phytophthora infestans* is a complex and adaptive eukaryotic lifeform that can spread and survive in multiple diverse forms through its life cycle, inside and outside its *Solanaceae* hosts (Fry, 2008; Fry et al., 2015). While mycelium growing on nutrient rich media appeared robust to the chemical treatments, we examined the impact of the individual volatiles exposure on both asexual forms that serve as infection vectors, namely sporangia and zoospores (Fry, 2008).

**FIGURE 3:**
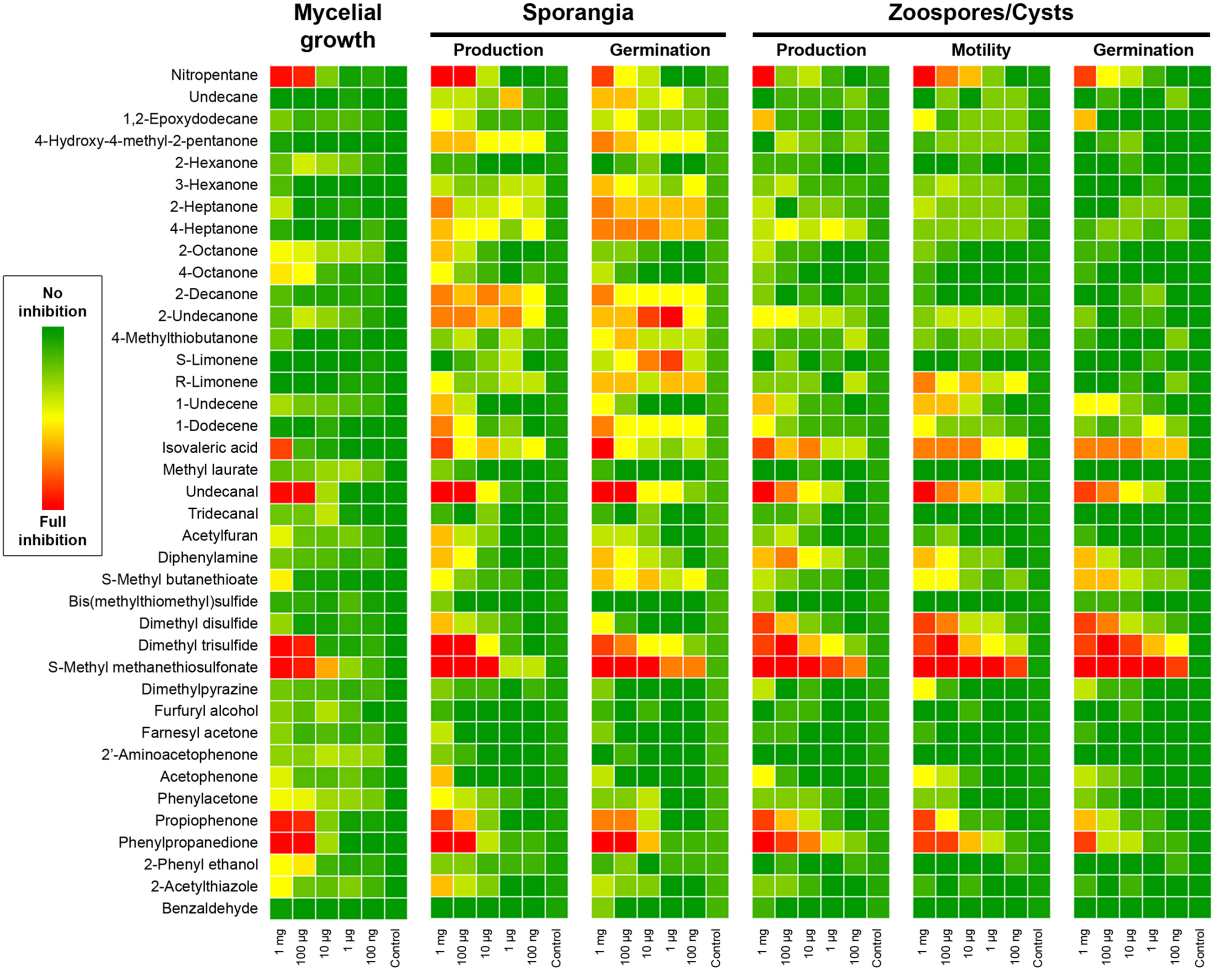
***Phytophthora infestans* mycelial growth, sporangiogenesis, zoosporogenesis, and spore development after exposure to *Pseudomonas* pure VOCs.** The heatmap color code represents the inhibitory effect obtained in each condition. For mycelial growth the percentage of total mycelium area compared to control is reported. Other parameters were scored on a 0–5 scale as follows: sporangia production, 0 = no sporangia in the field of view, 5 = 100% of control; sporangia germination, 0 = no germinated sporangia in the field of view, 5 = 100% of control; zoospore production, 0 = no zoospores in the field of view, 5 = 100% of control; Zoospore motility, 0 = no mobile zoospores, 5 = 100% of control; cystospore germination, 0 = no germinated cysts in the field of view, 5 = 100% of control. Results presented here are the average of two independent experiments with 4–5 replicates each.

We then assessed the ability of our *P. infestans* isolate to produce sporangia and discharge zoospores after the end of the mycelial growth period in presence of the pure volatiles. We also monitored the germination rate of both spore types and the motility of zoospores. Obviously chemicals that greatly impaired *Phytophthora* growth, nitropentane, isovaleric acid, undecanal, DMTS, MMTS, propiophenone and phenylpropanedione had a strong impact on sporangia production, and consequently on zoospore numbers (**Figure 3**). However, other compounds that did not greatly impede mycelial growth, such as the simple ketones 3-hexanone, 2- and 4-heptanone, 2-decanone and 2-undecanone, 4-hydroxy-methyl-2-pentanone as well as the alkenes 1-undecene and 1-dodecene significantly impacted sporangia production (**Figure 3**), suggesting that these mid-to-long hydrocarbon chains could specifically interfere with this process. At the highest amounts tested, diphenylamine and 2-acetylthiazole also decreased sporangial formation. The same set of chemicals also decreased the germination rate of sporangia, though this measurement does not depend on sporangia production, indicating that the spores may have been hindered during their developmental process or that non negligible amounts of the supplied VOCs were extracted during sample preparation. Based on the data displayed in **Figure 3**, limonene, 1-dodecene, and *S*-methylbutanethioate seem to have a specific effect on sporangia germination after mycelium exposure. Logically, the production of zoospores largely followed the pattern of sporangia production in terms of inhibition mediated by individual VOCs. None of the tested compounds seemed to specifically block this process. Further, zoospores released from sporangia freely swam in most treatments and exhibited no particular defects, with the notable exception of treatments of the mycelium with high amounts of MMTS, where a large number of zoospores underwent cell lysis. Nevertheless, exposure to 2-undecanone, limonene, 1-undecene, diphenylamine, *S*-methylbutanethioate, acetophenone, or phenylacetone during mycelial growth significantly decreased zoospore motility. Nitropentane, isovaleric acid, undecanal, as well as propiophenone and phenylpropanedione had also a strong negative impact on swimming in the highest treatments, while DMDS, DMTS, and MMTS showed the greatest inhibition. As for sporangia, the influence of pure compounds on the formation of germ tubes from encysted zoospores mostly resembled the effect on zoospore production. Taken together, these results suggest that *Pseudomonas* bVOCs have the potential to alter and impede *P. infestans* development, and that bioactive compounds mostly act similarly on the different lifeforms of the pathogen (mycelium, sporangia, zoospores) rather than specifically interfering with only one or the other.

### *Pseudomonas* Volatilome Encompasses Molecules Interrupting *P. infestans* Direct Sporangial Germination

The fungicides currently in use to constrain late blight spreading in potato crops vary in their activity against the different developmental stages in the life cycle of *Phytophthora* species, acting either against mycelial growth, zoospore release, zoospore motility, or germination of spores (Gisi and Cohen, 1996; Cohen and Gisi, 2007). Therefore, in order to select for *P. infestans* biocontrol biogenic volatiles, we focused this work on direct sporangia germination for the following reasons: (1) the *P. infestans* population shift observed since the 1980’s displays A2 mating type-dominated populations that tend to favor direct germination (Li et al., 2013; Fry et al., 2015), (2) sporangia are easily generated and collected in high yields, thus allowing larger screening efforts in the laboratory; (3) sporangia germination is easy to score and requires minimal equipment. We collected mature sporangia from control-grown *P. infestans* colonies and incubated them in serial dilutions of the test chemicals. After 24 h of treatment, about half of the chemicals tested showed inhibition of the germination process at 1 mg treatments (**Table 2**). However, the inhibitory effect rapidly dropped in most cases at lower drug amounts. Volatile species that demonstrated a significant inhibition potential (i.e., IC_50_ < 1 mg, reaching at least 30% inhibition and a *R*^2^ > 0.6) were 3-hexanone, 1-dodecene, isovaleric acid, *S*-methylbutanethioate, MMTS, furfuryl alcohol, acetophenone, phenylpropanedione, and 2-acetylthiazole (**Table 2**). Although 1-undecene and nitropentane treatments extrapolations did not meet these criteria, both molecules displayed satisfactory inhibitory power. Amongst these compounds, nitropentane, isovaleric acid and MMTS, as well as diphenylamine, were the only species to completely prevent the initiation of sporangia germination at 1 mg treatments. All other compounds displayed various degrees of germ tube elongation. Interestingly, the ketones 3-hexanone, 2-undecanone and, to a lesser extent, 2-decanone often led to decreased germ tube lengths and swellings or bursts of the growing germ tube tip, as well as ectopic initiation site (Supplementary Figure S4). This suggests that accumulation of these molecules specifically hinders the normal growth of the germ tube but not its initiation *per se*. We conclude that the sporangial germination process is widely sensitive to biogenic volatiles. Although the amounts employed in these experiments do probably not reflect the bVOCs quantities produced in the rhizosphere or phyllosphere in addition to the poor water solubility of some test chemicals, it seems reasonable to postulate that several individual volatiles contribute synergistically to the activity of the whole blend of bVOCs.

**Table 2:** *Phytophthora infestans* sporangia germination inhibition after exposure to pure VOCs.

	No constraints				Constrained fit Top = 100, Bottom= 0^a^	
	Bottom^a^	Top^a^	Rel. IC50 (mg)	*R*^2^	IC50 (mg)	*R*^2^
Nitropentane	30.68	98	6.054	0.7341	0.000252	0.4156
Undecane	87.97	∼103.4	∼6.271E-12	0.1019	5.771	-0.1492
1,2-Epoxydodecane	84.19	101.3	9.437E-7	0.1212	^∗^	^∗^
4-Hydroxy-4-methyl-2-pentanone	22.11	88.35	0.1844	0.4354	0.2635	0.3038
2-Hexanone	81.61	101.5	6.951E-5	0.132	2.818	-0.09947
**3-Hexanone**	**11.26**	**100.8**	**0.1289**	**0.6432**	**0.1837**	**0.6372**
2-Heptanone	41.46	102.1	0.06601	0.4721	0.5089	0.3916
4-Heptanone	35	108	0.03331	0.485	0.1637	0.3915
2-Octanone	53.09	96.4	0.01547	0.4561	0.7168	0.03963
4-Octanone	63.7	97.51	0.03694	0.1883	1.513	0.07603
2-Decanone	84.93	101.3	0.2728	0.07671	7.492	0.07136
2-Undecanone	81.67	101.4	4.226E-5	0.1358	2.818	-0.1261
4-Methylthiobutanone	90.44	102.8	2.505E-4	0.04701	7.544	-0.00575
*S*-Limonene	∼-11300	97.04	∼199.6	0.4006	0.7464	0.3854
*R*-Limonene	53.63	99.38	0.2099	0.1713	1.455	0.1585
1-Undecene	∼-14410	93.32	∼182.2	0.7917	0.3083	0.7284
**1-Dodecene**	**24.46**	**96.56**	**0.108**	**0.7615**	**0.2333**	**0.701**
**Isovaleric acid^†^**	**14**	**94.64**	**5.327E-4**	**0.81**	**0.000796**	**0.7574**
Methyl laurate	83.74	100.6	2.169E-5	0.08009	2.313	-0.00186
Undecanal	30.21	97.99	3.345E-5	0.7653	0.000142	0.4182
Tridecananl	46.49	∼98.06	∼8.653E-10	0.63	0.001213	-0.3044
Acetylfuran	33.66	98	9.805E-6	0.4782	9.77E-05	0.1851
Diphenylamine	-82.89	84.46	1.065	0.5583	0.1593	0.4402
***S*-Methylbutanethioate**	-**18.13**	**93.34**	**0.2622**	**0.7281**	**0.1404**	**0.7005**
Bis(methylthiomethyl)sulfide	-42.27	87.04	0.6047	0.5751	0.1619	0.4925
Dimethyl disulfide	^∗^	^∗^	^∗^	^∗^	^∗^	^∗^
Dimethyl trisulfide	46.05	94.39	0.08824	0.2538	0.6927	0.1751
***S*-Methyl methanethiosulfonate^†^**	**4.41**	**88.1**	**0.006435**	**0.7354**	**0.005417**	**0.6915**
Dimethylpyrazine	^∗^	^∗^	^∗^	^∗^	2.1E-11	-5.633
2-Dodecanol	^∗^	^∗^	^∗^	^∗^	^∗^	^∗^
**Furfuryl alcohol**	**8.759**	**94.98**	**0.2501**	**0.65**	**0.2742**	**0.6267**
Farnesyl acetone	82.46	101.2	7.91E-5	0.1591	4.674	-0.2519
2′-Aminoacetophenone	93.41	100.8	0.00656	0.01404	10.74	0.008978
**Acetophenone**	-**2.388**	**90.19**	**0.1245**	**0.6696**	**0.07803**	**0.6292**
Phenylacetone	89.14	100.5	2.18E-4	0.0382	13.66	-0.06405
Propiophenone	24.83	91.18	0.05952	0.5433	0.1185	0.4394
**Phenylpropanedione^†^**	**9.045**	**90.58**	**0.09959**	**0.6167**	**0.09691**	**0.5636**
2-Phenylethanol	^∗^	^∗^	^∗^	^∗^	^∗^	^∗^
**2-Acetylthiazole**	-**65.09**	**97.91**	**1.258**	**0.7521**	**0.4172**	**0.7421**
Benzaldehyde	82.16	98.91	0.001011	0.1005	3.161	-0.02778

*^a^% of P. infestans sporangial germination; ^†^Compounds considered efficient in both mycelial growth and sporangial germination inhibition; ^∗^Curve fitting did not converge. Grayed cells indicate S-containing compounds. Relevant non-linear regression parameters [Log(inhibitor) vs. Response] are derived from pharmacological assays. All test compounds were assayed at 1 mg, 100 μg, 10 μg, 1 μg, 100 ng per ml. In addition, DMDS, DMTS, and MMTS were tested at 10 ng per ml, n = 4. Curve fitting was performed with or without maximal constraints (top = 100% of P. infestans growth, bottom = 0% of P. infestans growth) to extrapolate IC_50_ and relative IC_50_ values for each compound, respectively. Bold indicates compounds displaying at least 30% inhibition, an IC_50_ below 1 mg and R^2^ > 0.6.*

### Sulfur-containing and Simple Ketones VOCs are *Bona Fide P. infestans* Inhibitors

**FIGURE 4:**
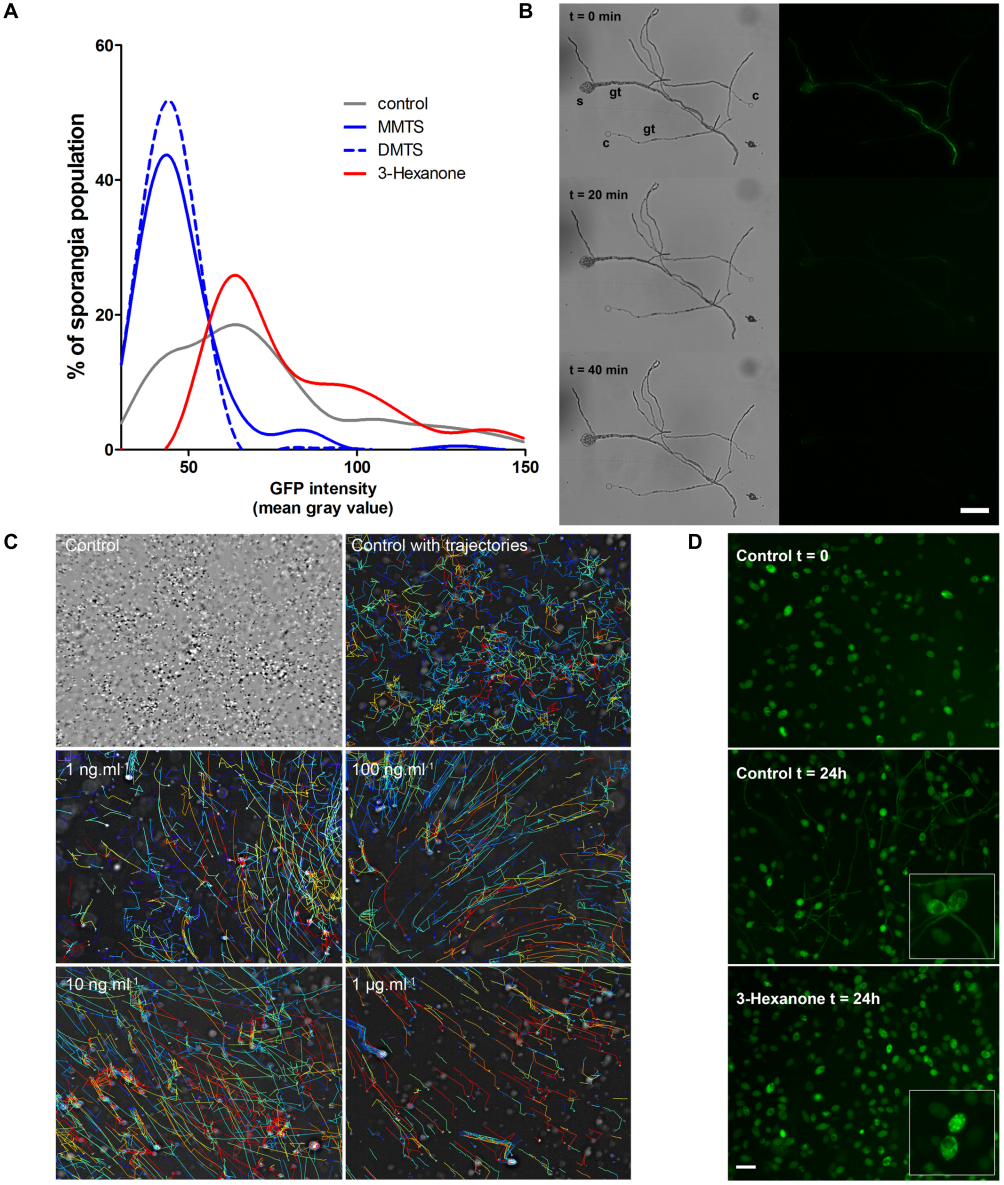
**(A)** Distribution of *P. infestans* 208 m^2^ sporangial population fluorescence intensity after exposure to selected single *Pseudomonas* VOCs. Representative curves shown here are from a single experiment using 10 μg treatments (*n* = 600–1500). Experiments were repeated three times with similar results. **(B)** 208 m^2^ sporangium (s) and cystospores **(C)** show a rapid GFP signal decrease after MMTS treatment. Gt, germ tube. Bar = 50 μm. **(C)** Single particle tracking of zoospores upon MMTS treatments. Upper-left image shows an overlay of individual time lapse frames. Every second frame was color-inverted to expose the zoospore movements. Computed velocities of individual zoospores trajectories are shown in a thermal color gradient, red = high and blue = low velocity. Note the straightening of the trajectory lines under MMTS treatments. The experiment was repeated at least three times with similar results. **(D)** Representative micrographs of *P. infestans* 208 m^2^ sporangia germinating in presence or absence of 3-hexanone. Inserts show higher magnification of the inner sporangia GFP signal. Bar = 50 μm.

Out of the forty single volatiles we tested, two compound groups retained our attention for their potent differential activity on *P. infestans* (**Figure 3** and **Table 2**): the sulfur-containing compounds DMDS, DMTS and MMTS and the simple ketones 3-hexanone, 2-decanone, and 2-undecanone. The first compounds seem able to block *P. infestans* growth and development while the latter specifically hinders sporangial germination when directly applied to the sporangia. We therefore investigated further the mode of action of DMTS, MMTS, and 3-hexanone using *P. infestans* 208 m^2^, a strain constitutively expressing the GFP fluorescent protein (Si-Ammour et al., 2003). With the aim to examine if the tested VOCs show sporicidal activities, we compared the GFP signal intensities of sporangial populations exposed to increasing amounts of volatiles for 24 h (**Figure 4A**). While control sporangia populations displayed a broad distribution of mean fluorescence signal intensities, ranging from close to background to highly fluorescent sporangia, both DMTS and MMTS treatments shifted the population fluorescence to low signals, presumably indicating that sporangia died during treatments. This hypothesis was further verified by treating ungerminated and germinated, GFP-expressing *Phytophthora* sporangia and cystospores with increasing amounts of these sulfur compounds. After 20 min of exposure with 1 mg MMTS, the GFP signal dramatically decreased in both spores and germ tubes (**Figure 4B**) and appeared practically abolished after 40 min without affecting the cellular structure while control treatments showed constant GFP signals over the same time frame. This suggests that MMTS directly blocked the spores’ cellular activity. In addition, propidium iodide cell viability tests confirmed the death of treated cells (results not shown). Since cell wall-free zoospores are recognized as being highly sensitive to exogenous chemicals (Judelson and Blanco, 2005; Chen et al., 2012) we tested the zoosporicidal potential of MMTS. We subjected fresh, freely swimming zoospore preparations to serial dilutions of MMTS and assessed their motility under the binocular. Representative zoospore trajectories obtained via computer-assisted single particle tracking are shown in **Figure 4C**. Surprisingly, MMTS treatments displayed a significant decrease of zoospore swimming at concentrations as low as 1 fg.ml^-1^, thus revealing a strong potency (data not shown). At highest concentrations (>1 μg.ml^-1^) MMTS immediately blocked zoospore motility followed by cell lysis. Moreover, during the course of these experiments carried out in 24-well plates, the volatile diffusion of MMTS from the high concentration wells was sufficient to inhibit completely zoospore motility in distant control wells.

Conversely, exposure of GFP-expressing sporangia to 3-hexanone led to a population shift toward sporangia with higher fluorescent signals that did not correlate with sporicidal effects (**Figure 4A**). At highest amounts (>10 μg.ml^-1^), 3-hexanone treatments triggered a reorganization of the inner sporangial GFP signal comparable to that of germinating sporangia although without germ tube production (**Figure 4D**). These observations, together with the abnormalities observed during sporangial germination described above (Supplementary Figure S4) seem to indicate that 3-hexanone is not sporicidal but rather directly interferes with the germination process of sporangia.

### *S*-Containing VOCs Protect Leaf Disk from Sporangia-mediated Tissue Invasion

**FIGURE 5:**
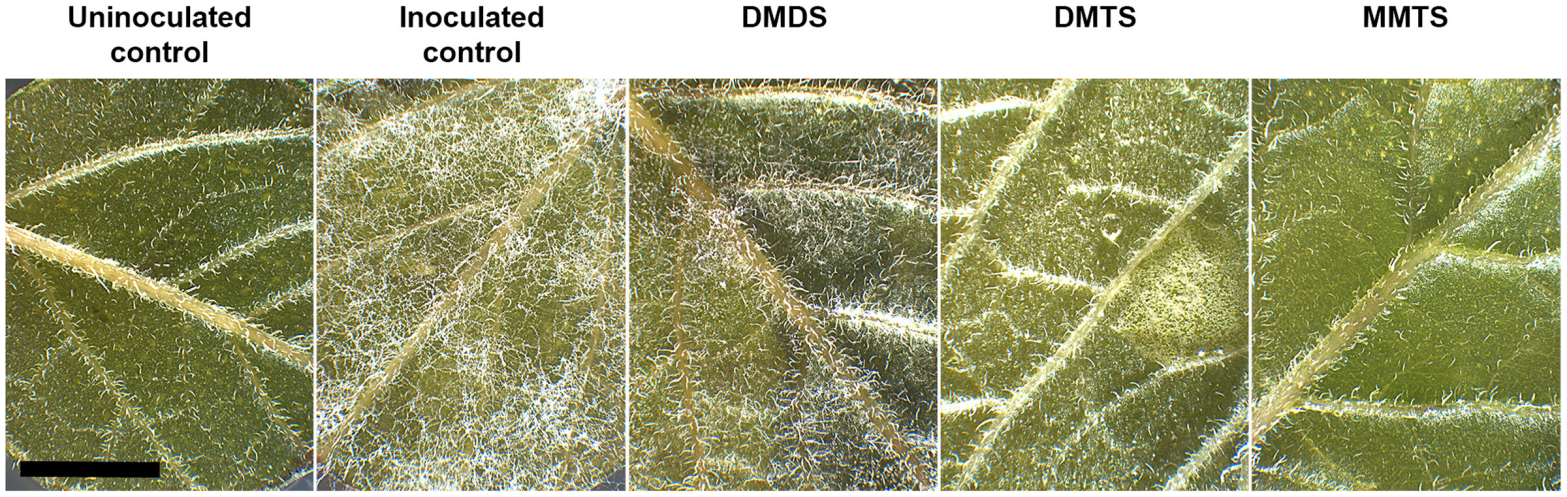
**Representative micrographs of VOC-treated potato leaf disK infection assays.** Note the complete absence of necrotic tissues or *P. infestans* sporangiophores in both DMTS and MMTS treatments. The experiment was repeated three times with similar results. Bar = 5 mm.

The ultimate goal of this work was to investigate whether biogenic volatiles produced by native potato-associated bacteria could be exploited to control late blight infection in planta. Consequently, we used our standardized laboratory leaf disk assay to assess the impact of pure VOCs on disease progression. Potato leaf disks inoculated with a sporangial suspension droplet were incubated for 8 days in presence or absence of 1 mg of DMDS, DMTS, or MMTS (**Figure 5**). At the end of the experiment, inoculated unexposed controls displayed strong symptoms with a dense mat of sporangiophores while treatments with the sulfur compounds reflected their respective inhibitory potential deduced from the experiments described above. At this amount, exposure to DMDS drastically reduced sporangiophore production and both DMTS and MMTS treatments prevented disease development. Regarding the facts that our leaf disk experimental design favors the direct sporangial germination in *P. infestans* infection, this suggested that both DMTS and MMTS treatments blocked this event. This assumption was further confirmed by microscopic observations (data not shown).

## Discussion

Late blight remains the most devastating potato disease worldwide and is commonly managed via recurrent applications of a wide range of systemic fungicides in conventional farming or of copper-based products in organic farming, respectively (Gachango et al., 2012; Olle et al., 2014). However, the selection pressure due to the increase in fungicide spraying frequencies in combination with the emergence of rapidly changing, recombinant pathogen populations first observed in the 1980s has brought the problem of resistance to the field (Zwankhuizen and Zadoks, 2002; Nowicki et al., 2012; Wang et al., 2012; Childers et al., 2015) and conventional late blight control has become tenuous. Likewise, the deleterious accumulation of copper and its toxicity for the soil ecosystems urgently calls for alternate, innovative solutions to fight *P. infestans* while preserving the environment (Dorn et al., 2007).

Many laboratories trying to pinpoint the molecules responsible for the observed activity of biogenic emissions of VOCs face the enormous complexity of the obtained compounds mixtures. In a previous work, we tested if the major component of the *Pseudomonas* volatilome, namely 1-undecene, had sufficient inhibitory power to hinder *P. infestans* growth and development (Hunziker et al., 2015). Although 1-undecene certainly contributes to the total activity of the whole volatile blend, the doses required to achieve significant *P. infestans* growth inhibition were very high. One would expect that the most potent chemical species should grant inhibitory effects even at very low amounts. Bacteria producing large amounts of the volatile respiratory inhibitor HCN or ammonia were already demonstrated to hold such antimicrobial properties (Voisard et al., 1989; Rudrappa et al., 2008; Hunziker et al., 2015). However, *Pseudomonas* strains devoid of HCN or ammonia synthesis pathways have also been reported to impede the growth and development of several fungal or fungal-like species (Trivedi et al., 2008; Athukorala et al., 2010; Elkahoui et al., 2015; Hunziker et al., 2015; Sheoran et al., 2015), which has stimulated increasing interest to further explore the bacterial volatilomes. Indeed, the growing knowledge on the chemical diversity of VOCs produced by bacteria (Schulz and Dickschat, 2007; Bos et al., 2013; Kanchiswamy et al., 2015) shed light on the latent discovery of new molecules that could contribute to the development of sustainable crop management strategies. Hence, our deep although not yet exhaustive screening effort for the anti-oomycete activities of individual volatile chemicals produced by plant-associated *Pseudomonas* strains demonstrated that the VOCs-mediated inhibition of *P. infestans* observed with the whole natural blends is probably not exclusively caused by specific single chemical species, but is most likely a result of the joint and possibly synergistic activities of several compounds. Although the repertoire of *Pseudomonas* VOCs contains very harmful substances toward *Phytophthora*, such as DMTS or MMTS, single active ingredients of the complex mixture are unlikely to serve as a molecular marker for biocontrol strain selection. Supplementary quantification of the amounts and effects of individual volatile products on *P. infestans* and the inhibitory effects of entire bouquets of bacterial strains could further help to statistically determine the contribution of discrete compounds to the synergistic inhibitory action of a given volatilome. Such robust multivariate analysis was already performed to discriminate bacterial species and strains based on their VOCs profiles (Thorn et al., 2011).

It is generally admitted that bVOCs mainly originate from background catabolism such as degradation of fatty acids, proteolysis and glycolysis (Schulz and Dickschat, 2007; Kai et al., 2009; Kanchiswamy et al., 2015). It is then not surprising that most strains investigated in this study, although genetically diverse among the *Pseudomonas* group, present similar VOCs profiles (**Figure 1**, Supplementary Figure S2). Grown in the same conditions, our test strains exhibited slight variations in the amounts of produced compounds that point to no obvious relationship between the composition of the volatilome and its overall inhibitory activity. However, close scrutiny of the enzymatic activities leading to the dynamic production of most potent volatile species could help understanding the collected experimental data. To that purpose, a comparative analysis of the plant-associated microbiome genomic data should be carried out to further detail the presence or absence of key genetic traits that grant efficient VOCs toolsets to individual bacterial strains or isolates. In particular, monitoring the expression of pivotal genes or proteins under different relevant growth conditions (Omasits et al., 2013) could provide useful differential expression information about the activity of selected pathways that lead to volatile production, as well as the dynamics of VOCs biogenesis and its occurrence *in natura*. Manipulations of the *Pseudomonas* critical VOCs-related genes would as well detail the individual contribution of single compounds, as demonstrated for plant-growth promoting, indole-producing rhizobacteria (Bailly et al., 2014). Finally, this knowledge, together with a continuous flow of reports of the identification of novel volatilome constituents could lead to the establishment of synthetic volatile combinations mimicking the natural blends. Such synthetic mixtures pave the way for their systematic pharmacological testing to investigate the putative synergistic effects of such complex signals. This approach has been successfully employed with VOCs originating from the fern endophytic fungus *Nodulisporium* sp., suppressing the growth of various plant fungal and oomycetes pathogens at low concentrations (Riyaz-Ul-Hassan et al., 2013).

Many studies, including our work, have reported the inhibition of a growing number of distinct plant pathogens through bacterial emissions in dual assays (Rengel and Marschner, 2005; Vespermann et al., 2007; Athukorala et al., 2010; Ting et al., 2011; Velazquez-Becerra et al., 2011; Effmert et al., 2012; Yuan et al., 2012; Groenhagen et al., 2013; Tenorio-Salgado et al., 2013; Wang et al., 2013; Contreras-Cornejo et al., 2014; Audrain et al., 2015; Hunziker et al., 2015), thus describing an experimental setup where bacteria grew on rich media in the physical presence of the target species. However, for technical reasons, the chemical identification of volatile compounds is generally performed in absence of the pathogen. Still, the possibility that bacteria respond to the target species should not be excluded and assessments of the expression levels of genes implicated in the production of VOCs in the presence of the pathogen should be performed. Such experiments are currently under investigation in our laboratory. Furthermore, the experimental data demonstrating a clear role of VOCs in the direct biocontrol of plant pathogens outside of the controlled, *in vitro* laboratory setting is lacking to date. While the prospective sum of work has underlined the potential of volatiles in mediating benefits in plant health and fitness (Bailly and Weisskopf, 2012; Farag et al., 2013; Kanchiswamy et al., 2015), the concrete contribution of bVOCs to direct plant protection requires further study. As an example, the nutrient-rich rhizosphere hosts a vast microbial diversity and is assumed to offer enough metabolite variety to contribute to volatile production (Morgan et al., 2005; Rengel and Marschner, 2005; Bulgarelli et al., 2013). However, the cellular populations growing on the root surface are by far smaller than those used for chemoprofiling and one could expect that each individual microbial species adds to the complexity of the total volatilome expressed *in situ*. Blom et al. (2011a) clearly demonstrated that biogenic emissions primarily depend on the composition of feeding media, thus inferring that the volatiles profile of a given strain grown in Petri dishes would qualitatively and quantitatively differ from the profiles occurring in the natural plant-bacteria association. The same study also convincingly reported that bacteria have the ability to generate complex volatilomes even in very limited nutrient conditions (Blom et al., 2011a). Still, the experimental design that could precise the identity and amounts of VOCs produced on the root surface is yet to be established.

We report here, beyond the *P. infestans* mycelial growth inhibition, the negative impact of a dozen of *Pseudomonas* volatiles on the normal spore development, and clear sporicidal activity for four common components of the VOCs blend. Given the sporangia germ tube malformations triggered by some of the tested ketones and the general inhibitory potential of the compounds assayed against both sporangia and zoospores, it seems reasonable to assume that the volatiles from *Pseudomonas* strains that we isolated from the potato plant could participate in a supplementary defense line against *Phytophthora* infection. As discussed above, the prerequisites are in one hand that the synthesis of these active compounds occurs at local levels and in the other hand that sufficient amounts should be produced to reach inhibitory conditions. The mucilage surrounding the root cap, the porous nature of soil and the stomatal chamber space offer space-restricted niches where accumulation of VOCs can happen and where both bacteria and the pathogen can interact. The nascent concept of biofumigation (Matthiessen and Shackleton, 2005; Goates and Mercier, 2009; Morales-Rodriguez et al., 2012) envisages the application of natural bioactive compounds such as the plant-growth promoting, sulfur-containing DMDS (Meldau et al., 2013), already in use as suppressive soil fumigant in agriculture (Auger et al., 1989; Van Wambeke et al., 2009), via the enrichment of VOC-producing microorganisms to target pathogens. Therefore, the isolation, characterization, selection and stable reintroduction of native plant-associated bacteria into potato crops promises an efficient and sustainable strategy to manage late blight at low costs. Alternatively, the natural origin of potent inhibitors identified in the bacterial volatilome, like DMTS or MMTS, could also lead to different organic farming strategies as they can be readily extracted from *Cruciferae* and *Liliaceae* species (Nakamura et al., 1996; Kyung and Fleming, 1997).

## Author Contributions

AB and LW designed the research; AB, MD, PP, and AV performed experiments; AB, AV, TB, and MD analysed the data; AB wrote the manuscript with help from LW, CA, and TB.

## Conflict of Interest Statement

The authors declare that the research was conducted in the absence of any commercial or financial relationships that could be construed as a potential conflict of interest.

## Footnotes

1 http://www.ncbi.nlm.nih.gov/genbank/

2 imagej.nih.gov

3 http://www.graphpad.com/quickcalcs/
