# Supplementary material for: Volatile Organic Compounds from Native Potato-associated Pseudomonas as Potential Anti-oomycete Agents
Source: Front Microbiol. 2015 Nov 23;6:1295. doi: 10.3389/fmicb.2015.01295 (PMC4655239; doi:10.3389/fmicb.2015.01295)
Supplement: Supplementary file 1 [file Data_Sheet_1.PDF]

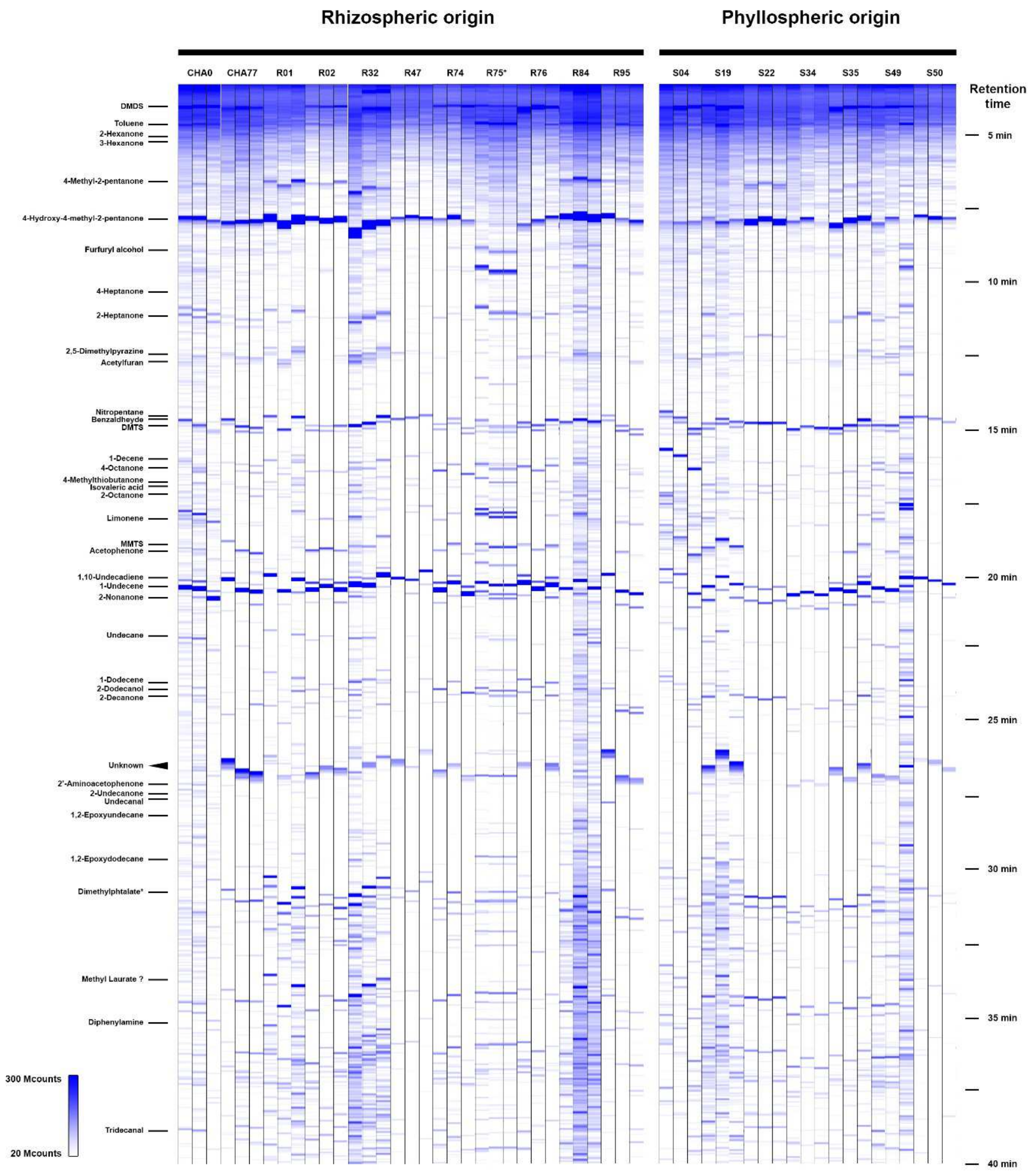

Supplementary figure 1. Chemoprofiles of volatile organic compounds emitted by potato-associated *Pseudomonas*. Three representative gas chromatography spectra for each strain are ordered according to their isolation origin. Identified compounds and related retention times are indicated on the left. *R75 was not identified as a *Pseudomonas* strain.

a

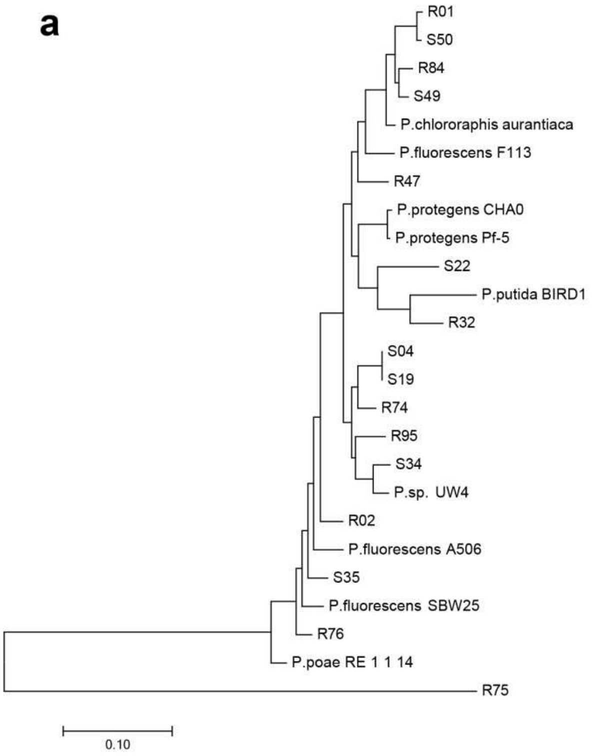

b

Reference Strain	Accession Number
Pseudomonas putida BIRD-1	NC_017530
*Pseudomonas poae RE*1-1-14*	NC_020209
Pseudomonas protegens CHAO	NC_021237
Pseudomonas protegens Pf-5	NC_004129
Pseudomonas sp. UW4	NC_019670
Peudomonas fluorescens SBW25	NC_012660
Pseudomonas fluorescens A506	NC_017911
Pseudomonas fluorescens F113	NC_016830
Pseudomonas chlororaphis strain EA105	JSFK01000001

c

Gene	Nucleotide sequence	GenBank accession number	Gene	Nucleotide sequence	GenBank accession number
**16s rRNA**	SUB1131374 seqID1	KT890299	**rpoB**	BankIt1862473 seqID1	KT890314
	SUB1131374 seqID2	KT890300		BankIt1862473 seqID2	KT890315
	SUB1131374 seqID3	KT890301		BankIt1862473 seqID3	KT890316
	SUB1131374 seqID4	KT890302		BankIt1862473 seqID4	KT890317
	SUB1131374 seqID5	KT890303		BankIt1862473 seqID5	KT890318
	SUB1131374 seqID6	KT890304		BankIt1862473 seqID6	KT890319
	SUB1131374 seqID7	KT890305		BankIt1862473 seqID7	KT890320
	SUB1131374 seqID8	KT890306		BankIt1862473 seqID8	KT890321
	SUB1131374 seqID9	KT890307		BankIt1862473 seqID9	KT890322
	SUB1131374 seqID10	KT890308		BankIt1862473 seqID10	KT890323
	SUB1131374 seqID11	KT890309		BankIt1862473 seqID11	KT890324
	SUB1131374 seqID12	KT890310		BankIt1862473 seqID12	KT890325
	SUB1131374 seqID13	KT890311		BankIt1862473 seqID13	KT890326
	SUB1131374 seqID14	KT890312		BankIt1862473 seqID14	KT890327
	SUB1131374 seqID15	KT890313		BankIt1862473 seqID15	KT890328
**gyrB**	BankIt1861640 seqID1	KT890284	**rpoD**	BankIt1862550 seqID1	KT890329
	BankIt1861640 seqID2	KT890285		BankIt1862550 seqID2	KT890330
	BankIt1861640 seqID3	KT890286		BankIt1862550 seqID3	KT890331
	BankIt1861640 seqID4	KT890287		BankIt1862550 seqID4	KT890332
	BankIt1861640 seqID5	KT890288		BankIt1862550 seqID5	KT890333
	BankIt1861640 seqID6	KT890289		BankIt1862550 seqID6	KT890334
	BankIt1861640 seqID7	KT890290		BankIt1862550 seqID7	KT890335
	BankIt1861640 seqID8	KT890291		BankIt1862550 seqID8	KT890336
	BankIt1861640 seqID9	KT890292		BankIt1862550 seqID9	KT890337
	BankIt1861640 seqID10	KT890293		BankIt1862550 seqID10	KT890338
	BankIt1861640 seqID11	KT890294		BankIt1862550 seqID11	KT890339
	BankIt1861640 seqID12	KT890295		BankIt1862550 seqID12	KT890340
	BankIt1861640 seqID13	KT890296		BankIt1862550 seqID13	KT890341
	BankIt1861640 seqID14	KT890297		BankIt1862550 seqID14	KT890342
	BankIt1861640 seqID15	KT890298		BankIt1862550 seqID15	KT890343

Supplementary figure 2: a) Phylogenetic tree of the sixteen candidate Pseudomonas sp. strains and selected reference strains. The tree was generated with the MEGA software (Kumar *et al.*, 2008), using 4 concatenated housekeeping genes (in the order 16srRNA, gyrB, rpoD, rpoB) as input sequences, as described (Gomila *et al.*, 2015). Nine reference *Pseudomonas* strains were used in addition to the 16 strains isolated from the rhizosphere and phyllosphere of potato plants, which exhibited differential protection against infection with P. infestans. The bars in the dendogram indicate mean average base substitutions between the sequences. The greater the difference in the lengths of the bars between two species, the higher was the substitution rate between the sequences. The scale bar indicates 0.10 substitutions per nucleotide position. **b)** Reference *Pseudomonas* strains were selected from the NCBI database of complete genomes; accession numbers of the downloaded reference sequences are given and **c)** gene sequences generated in this study were deposited to GeneBank under the accession number reported here.

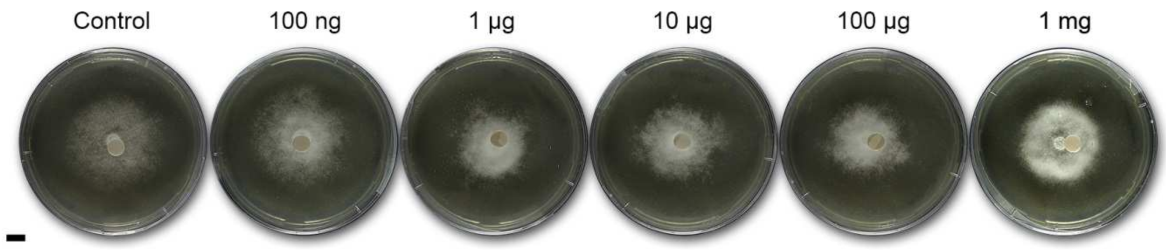

Supplementary figure 3. *P. infestans* mycelium phenotype triggered after exposition to 2-Undecanone. Note the densification of the mycelial mat. The experiment was repeated 3 times with similar results. Bar = 1 cm.

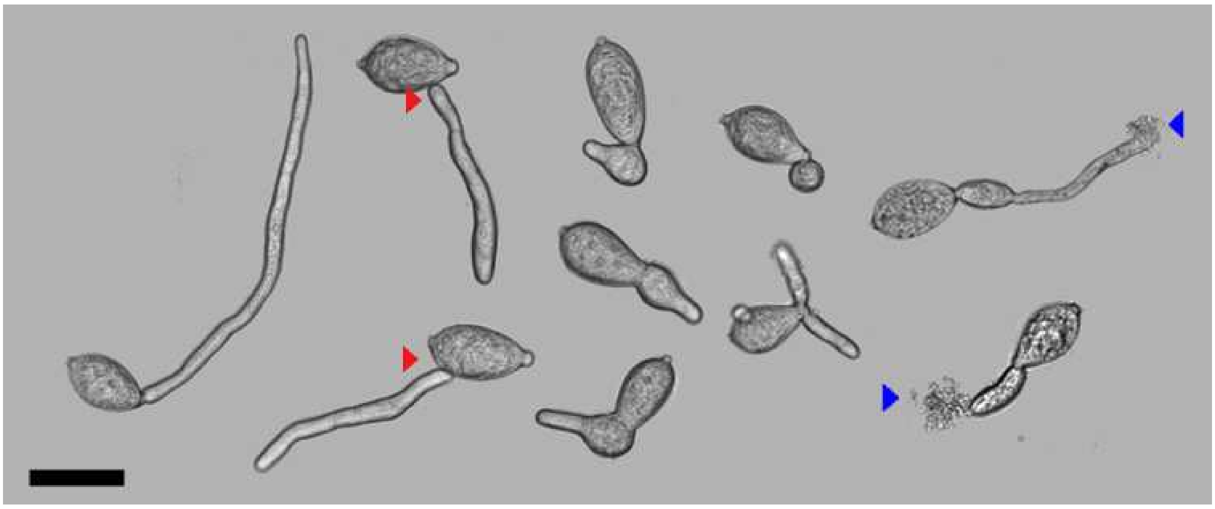

Supplementary figure 4. Representative sporangial germination defects after exposition to the ketones 3-hexanone, 2-decanone or 2-undecanone. Left, control germinating sporangium after 24 hours. Red arrows indicate ectopic germ tube initiation sites. Blue arrows indicate germ tube tip bursts. Bar = 40 µm.

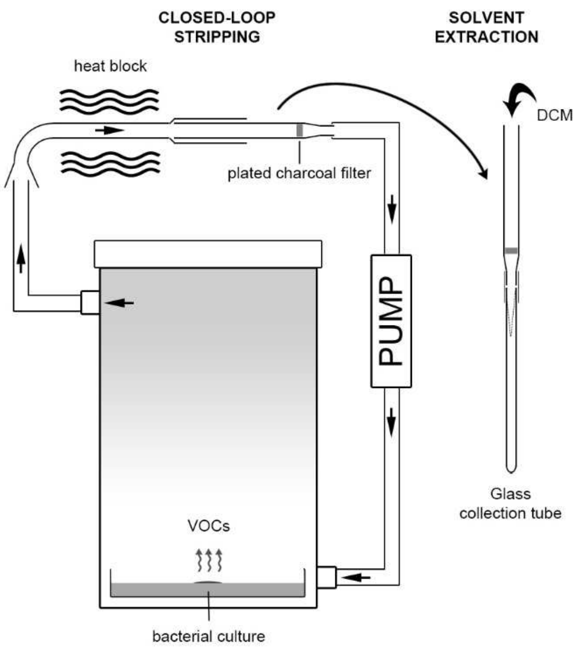

Supplementary figure 5. Modified CLSA apparatus for bacterial VOCs collection. Arrows indicate the air flow direction. The total volume of the PTFE chamber is 100 ml. DCM, dicholoromethane.
